# Mimes of the past: Eocene midges of the tribe Pseudochironomini (Chironomidae, Diptera) reveal their peculiarities

**DOI:** 10.1371/journal.pone.0295841

**Published:** 2023-12-27

**Authors:** Marta Zakrzewska, Trond Andersen, Wojciech Giłka

**Affiliations:** 1 Department of Invertebrate Zoology and Parasitology, Faculty of Biology, University of Gdańsk, Gdańsk, Poland; 2 Department of Natural History, University Museum of Bergen, University of Bergen, Bergen, Norway; Nanjing Agricultural University, CHINA

## Abstract

This is the first study focused on Eocene dipterans of the tribe Pseudochironomini (subfamily Chironominae, family Chironomidae), based on unique materials from Baltic amber. Two new genera and three new species: ***Eomicromimus* gen. nov.** with ***Eomicromimus polliciformis* sp. nov.** and ***Eomicromimus serpens* sp. nov.**, and ***Eoriethia* gen. nov.** with ***Eoriethia ursipes* sp. nov.** are presented. The systematic position of the new taxa is discussed, and an amended key to the identification of adult males of extinct and extant Pseudochironomini genera is provided. The presented analysis of the morphology of the tribe’s fossil members allowed us to verify the concepts regarding the origin/homology of male diagnostic structures crucial in defining new taxa, their phylogeny, and to consolidate the terminology used in chironomid research. A new habitual name for Chironomidae, “mime midges”, is also proposed.

## Introduction

With approximately 7 500 species, 550 genera and 12 subfamilies, Chironomidae is the largest dipteran family, but still only half of the world’s species are known at best [1, 2]. The Chironominae, which is probably the largest chironomid subfamily, is divided into four tribes, among which the Pseudochironomini is far from being well explored, both in terms of species diversity, understanding their morphology, and terminology used in diagnostics. Contrary to its sister tribe Tanytarsini, which has been extensively studied in recent years (i.a. [3–6]), so far only a couple of studies have dealt with fossil Pseudochironomini. These chironomids are known to have appeared no later than at the turn of the Early to Late Cretaceous [7], and prior to this study has been known from eight genera, including two extinct ones. The oldest fossil genus of the tribe Pseudochironomini, which is also the oldest Chironominae known to science, is the monotypic *Palaeocentron* Giłka, Zakrzewska, Lukashevich & Cranston, 2021, evidenced to exist in “mid-Cretaceous” (amber from Kachin, Myanmar; ~100 Mya). The second genus, *Mesoacentron* Giłka, Zakrzewska, Lukashevich & Cranston, 2021, comes from the Late Cretaceous Taimyr amber (Russia; ~84 Mya), thus being quite younger [7]. The remaining six genera are extant, including *Megacentron* with a sole fossil species of *M*. *eocenicus* Doitteau & Nel, 2007, reported from the Eocene Oise amber (France; ~53 Mya) [8].

Chironomidae are habitually called non-biting midges, and their English name became fixed as an antonym to biting midges, Ceratopogonidae, excluded from the Chironomidae into the separate family a century ago. However, the scientific name, Chironomidae, was originally most likely intended to emphasise the characteristic movements performed by the strongly elongated forelegs of imagines, since the translation of the Greek verb "*cheironomo*" (*χειρονομώ*) is "gesticulate", the Latin adjective "*chironomos*" is translated as "pantomimic", and the noun "*chironomon*"—as "someone playing pantomime or pretending to be someone" [9, 10]. The name "mime midges" seems thus more appropriate than "non-biting midges", which the latter name in fact could fit many other truly non-biting dipterans apart from the Chironomidae, whose mouthparts in some groups were (extinct taxa), and still are adapted for biting [11–14]. The behaviour of chironomids and the position of their long forelegs spread out to the sides or forward, strained, quavering, or moving in different directions, is often stopped in time in specimens embedded in fossil resins, thus we decided to keep it also in a name of one of the genera described here, that means “Eocene tiny mime”. What the Eocene mime midges show is a peculiarity of their morphology and diagnostic structures that we try to define below.

## Material and methods

### Fossil specimens and morphological analysis

Four fossil specimens with inventory numbers: CCHH 93–1, CCHH 93–4, CCHH 1754–5a and CCHH 1754–13, studied in this article are inclusions preserved in pieces of the Eocene Baltic amber (Gulf of Gdańsk, Poland) from the collection of Christel and Hans Werner Hoffeins (CCHH) of Hamburg, Germany. The types are booked to be deposited at the Senckenberg Deutsches Entomologisches Institut (SDEI), Müncheberg, Germany, where they will be easily accessible to all interested parties. No permits were required for the described study, which complied with all relevant regulations.

The amber pieces were ground and polished, so that the inclusions and their diagnostic structures could be examined at high magnification and photographed. Owing to the fragile nature of the amber, some pieces examined were embedded in artificial epoxy resin. A piece labelled CCHH 1754–13 was further treated to gain visibility of hypopygial area by hand-filling the space between amber layers with an epoxy resin.

Specimen dimensions are given in micrometres, except for the total body length (in millimetres, rounded off to the second decimal place). The body and wing lengths were measured from the antennal pedicel to the end of the gonostylus and from the arculus to the tip, respectively. The lengths of leg segments and palpomeres were rounded to the nearest 5 μm. The antennal, leg and venarum ratios were calculated to the second digit after the decimal point. Abbreviations of the morphological terminology used in the article are after Sæther [15] and Cranston [16]), and presently supplemented. Head: AR, antennal ratio; fm_1_–fm_13_, flagellomeres 1–13. Thorax chaetotaxy: Ac, acrostichal setae or acrostichals; Dc, dorsocentrals; Pa, prealars; Scts, scutellars. Wing venation: C, costa; FCu, cubital fork; M_1+2_, medius 1+2; R_1_–R_4+5_, radius 1–4+5; RM, radius-medius crossvein; Sc, subcosta; VRCu, RM to FCu length ratio. Legs: fe, femur; LR, leg ratio; p_1_–p_3_, pair of legs 1–3; ta_1_–ta_5_, tarsomeres 1–5; ti, tibia. Hypopygium: dl, dorsal lobe of superior volsella; IVo, inferior volsella; IVo aml, anteromedian lobe of inferior volsella; PVo, pseudovolsella; SVo, superior volsella; SVo al, anterior lobe of superior volsella; SVo pl, posterior lobe of superior volsella; vl, ventral lobe of superior volsella. Photographs were taken using a Leica M205 A and PZO Biolar SK14 microscopes with a Sony NEX-3N digital camera. The images were compiled using the Helicon Focus 8 image stacking software.

### Nomenclatural acts

The electronic edition of this article conforms to the requirements of the amended International Code of Zoological Nomenclature, and hence the new names contained herein are available under that Code from the electronic edition of this article. This published work and the nomenclatural acts it contains have been registered in ZooBank, the online registration system for the ICZN. The ZooBank LSIDs (Life Science Identifiers) can be resolved and the associated information viewed through any standard web browser by appending the LSID to the prefix “http://zoobank.org/”. The LSID for this publication is: urn:lsid:zoobank.org:pub:26BD474E-5364-4842-B0AB-B97804F6B415. The electronic edition of this work was published in a journal with an ISSN, and has been archived and is available from the following digital repositories: PubMed Central, LOCKSS, Knowledge Base of the University of Gdańsk, BORA (Bergen Open Research Archive).

## Results and discussion

### Systematics: New taxa

Family: Chironomidae Newman, 1834

Subfamily: Chironominae Newman, 1834

Tribe: Pseudochironomini Sæther, 1977

***Eomicromimus* Giłka, Zakrzewska *et* Andersen, gen. nov.** urn:lsid:zoobank.org:act:E0462D30-521D-4777-8CAA-ABB74F2E80AC

(Figs 1–6).

**Fig 1:**
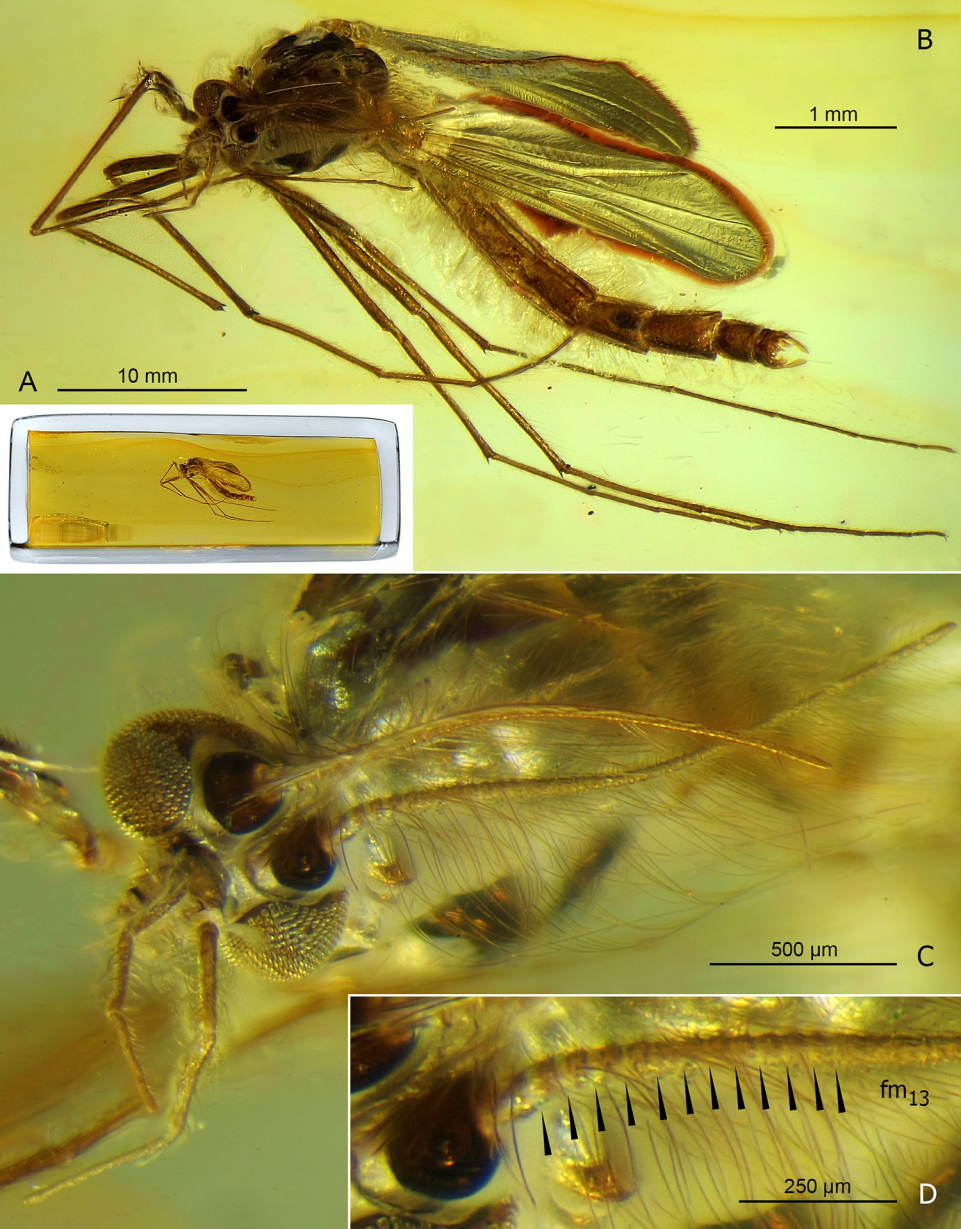
*Eomicromimus polliciformis* gen. *et* sp. nov., adult male, holotype (*CCHH 93–1*, Eocene Baltic amber). (A) Inclusion in amber embedded in epoxy resin. (B) Habitus. (C) Head. (D) Proximal part of antenna (arrowheads indicate borders between flagellomeres fm_1_–fm_13_).

**Fig 2:**
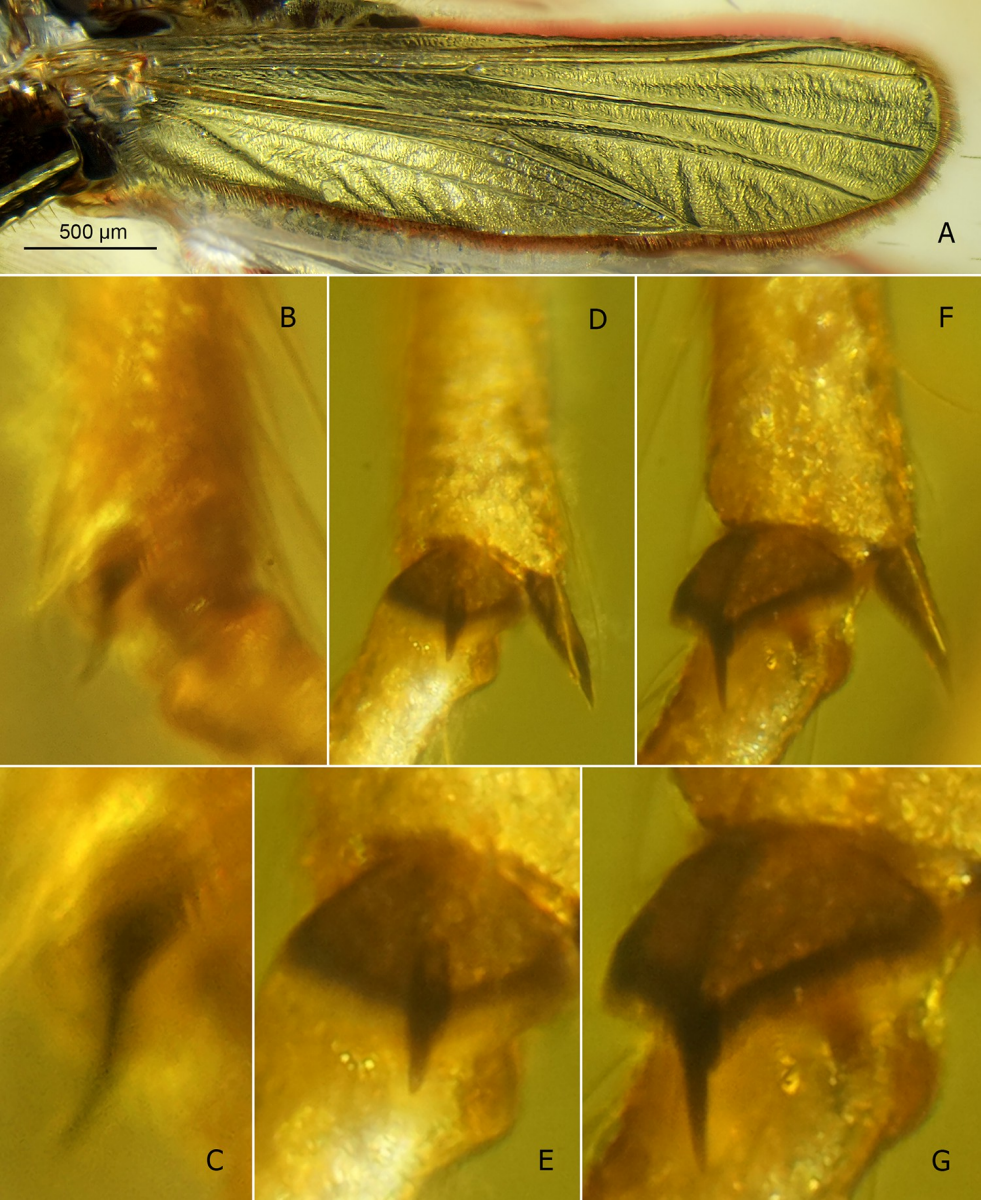
*Eomicromimus polliciformis* gen. *et* sp. nov., adult male, holotype (*CCHH 93–1*, Eocene Baltic amber). (A) Wing. (B–G) Tibial combs and spurs of fore (B, C), mid (D, E) and hind leg (F, G); C, E, G magnified ca. twice relative to B, D, F, respectively.

**Fig 3:**
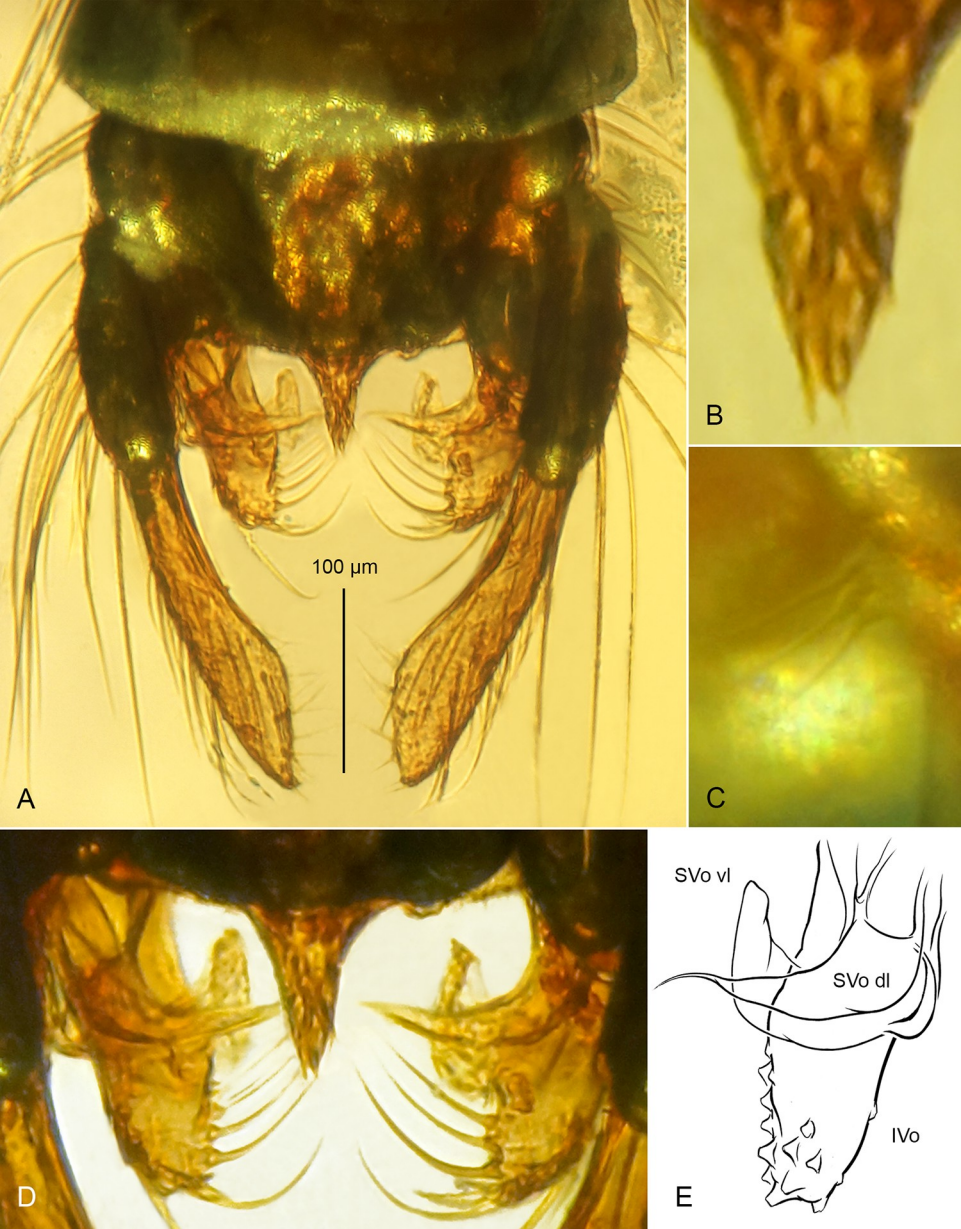
*Eomicromimus polliciformis* gen. *et* sp. nov., adult male, holotype (*CCHH 93–1*, Eocene Baltic amber). (A) Hypopygium in dorsal aspect. (B) Subapical paired structure of anal point magnified. (C) Pseudovolsella. (D) Anal point and volsellae magnified. (E) Volsellae on drawing: superior volsella (SVo) with its dorsal lobe (SVo dl) and ventral lobe (SVo vl), inferior volsella (IVo).

**Fig 4:**
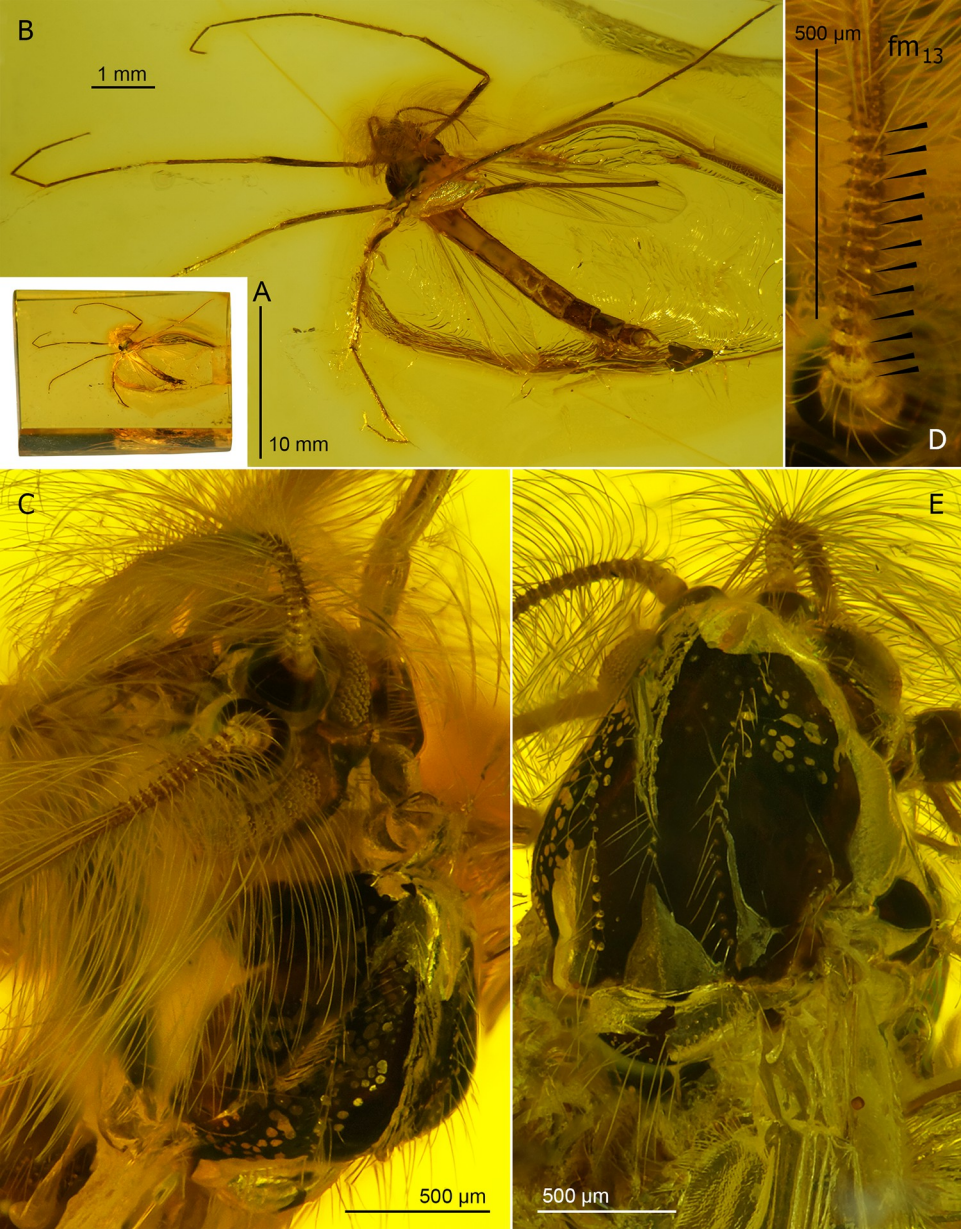
*Eomicromimus serpens* gen. *et* sp. nov., adult male, holotype (*CCHH 1754–13*, Eocene Baltic amber). (A) Inclusion in amber. (B) Habitus. (C) Head. (D) Proximal part of antenna (arrowheads indicate borders between flagellomeres fm_1_–fm_13_). (E) Thorax in dorsal aspect, and its chaetotaxy.

**Fig 5:**
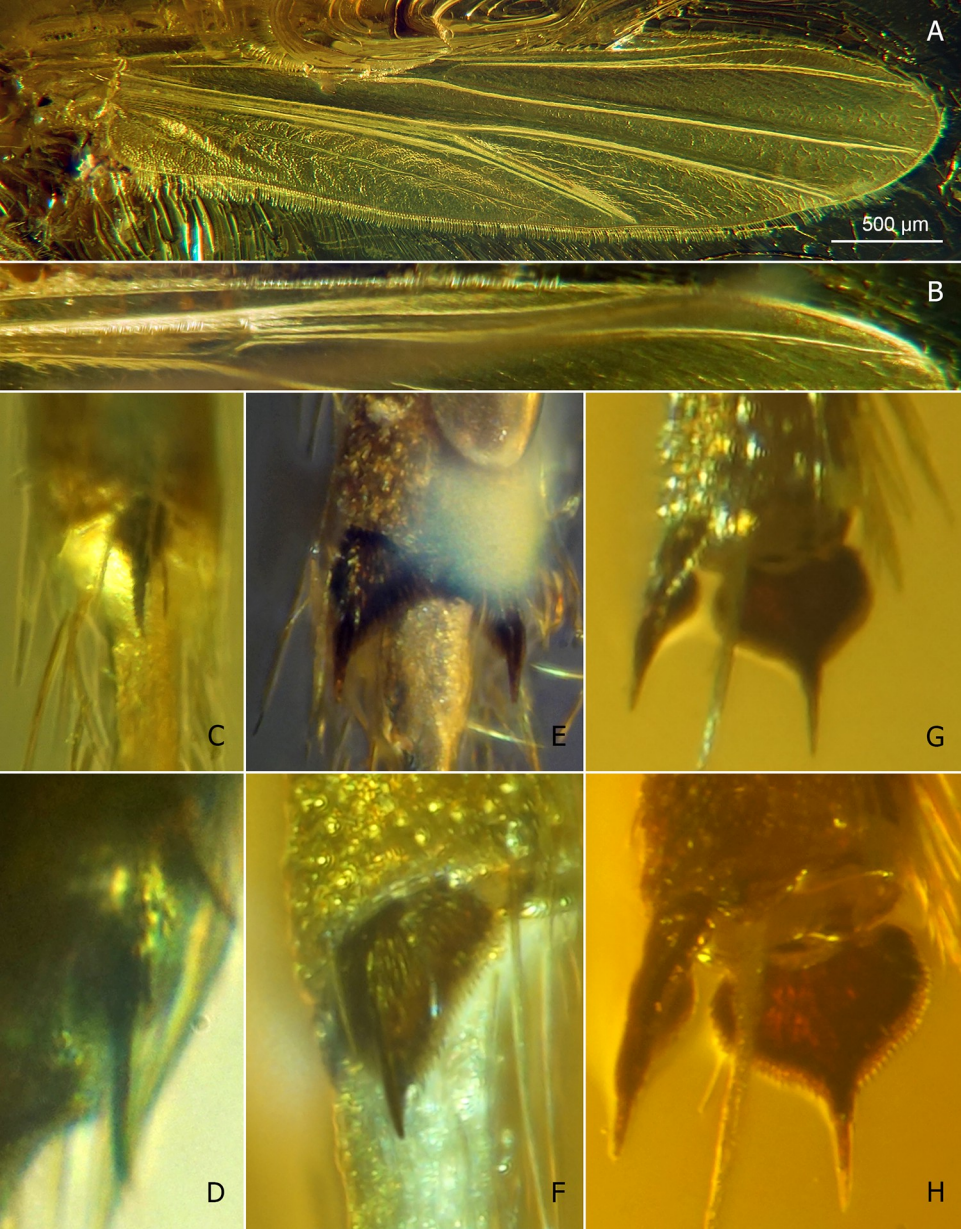
*Eomicromimus serpens* gen. *et* sp. nov., adult male, holotype (*CCHH 1754–13*, Eocene Baltic amber). (A, B) Wing and arrangement of veins in anterior area magnified. (C–H) Tibial combs and spurs of fore (C, D), mid (E, F) and hind leg (G, H); D, F, H magnified ca. twice relative to C, E, G, respectively.

**Fig 6:**
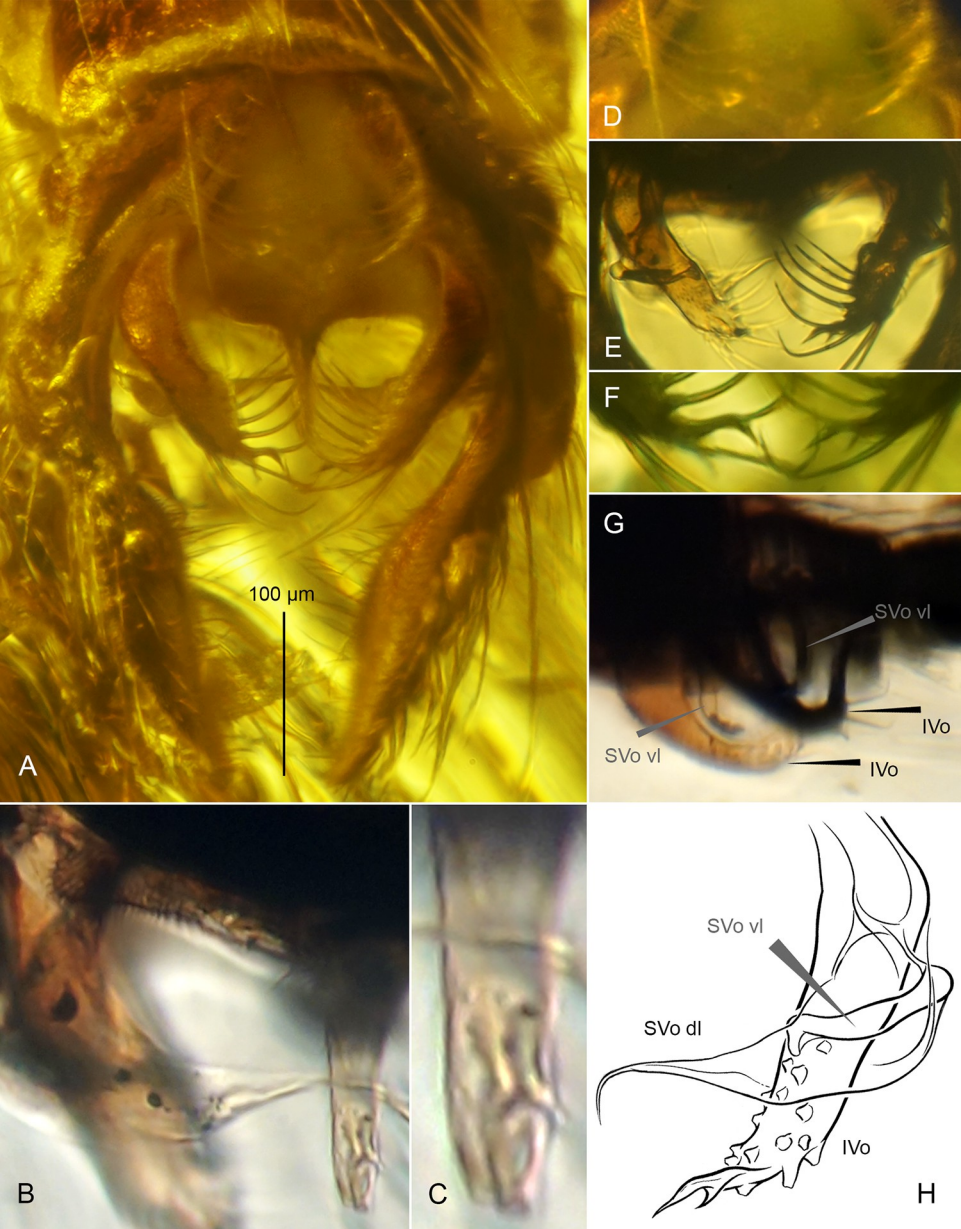
*Eomicromimus serpens* gen. *et* sp. nov., adult male, holotype (*CCHH 1754–13*, Eocene Baltic amber). (A) Hypopygium in ventral aspect. (B) Posterolateral margin of anal tergite (shoulder), superior volsella and anal point in dorsal aspect. (C) Subapical paired structure of anal point magnified. (D) Pseudovolsella. (E) Arrangement of volsellae. (F) Apices of inferior volsellae magnified. (G) Ventral lobes of superior volsellae (SVo vl) and inferior volsellae (IVo) in lateral aspect. (H) Volsellae on drawing: superior volsella (SVo) with its dorsal lobe (SVo dl) and ventral lobe (SVo vl), inferior volsella (IVo).

**Type species:** *Eomicromimus polliciformis* Giłka, Zakrzewska *et* Andersen, **sp. nov.** (by present designation).

**Derivation of the name:** The genus is named with reference to the strongly elongated forelegs characteristically moved by chironomids; the name derived from the words: Eocene (*Eo-*), tiny (*-micro-*), mime/actor (in Latin, *mimus*; see also Introduction).

**Generic diagnosis:** Eyes bare. Antenna with 13 flagellomeres. Wing squama small, with several setae. Anal tergite of hypopygium with posterolateral margins angulate, forming distinct shoulders. Anal point well-developed, with peculiar, paired structure subapically. Pseudovolsella in the form of merged setal tubercles. Superior volsella bilobed: dorsal lobe with broad base and rounded posterolateral margin, evenly tapering to an elongated distal part bearing filiform tip; ventral lobe variably shaped, but always with arcuate base forming a connection with the dorsal lobe. Digitus, true median volsella and pars ventralis absent.

***Eomicromimus polliciformis* Giłka, Zakrzewska *et* Andersen, sp. nov.** urn:lsid:zoobank.org:act:464A86C2-7FB7-48CB-AB23-321DE2D6B96F

(Figs 1–3)

**Derivation of the name:** In reference to a stout, thumb-shaped ventral lobe of the hypopygial superior volsella.

**Type material:** Holotype, *CCHH 93–1*: adult male (tarsus of right midleg missing) preserved in a 20 × 6.5 × 5 mm piece of Eocene Baltic amber enclosed in a 22.5 × 8 × 7.5 mm cubicoid piece of epoxy resin (Fig 1A and 1B).

**Diagnosis:** Macrotrichia present only on wing margin. Gonostylus arched, spatulate, broadened in distal part. Anal point triangular, bearing posteriorly directed scale-like spines on its dorsal surface. Pseudovolsella formed by three merged tubercles. Ventral lobe of superior volsella robust, with proximal part projecting medially, distinctly bent at mid-length and directed anteriorly, its apex thumb-shaped. Inferior volsella stocky, with broad apex.

**Description:** [adult male (n = 1, holotype)]

Total body length: 5.60 mm; wing length: 3050 μm.

**Head** (Fig 1C and 1D): Eyes bare, kidney-shaped, with well-developed dorsomedian extensions. Frontal tubercles not observed. Antenna with 13 distinctly separated flagellomeres (Fig 1D), AR 1.83, plume fully developed. Length of palpomeres 2–5: ~140 μm, 230 μm, 250 μm, 365 μm. Clypeus with at least 8 fine setae.

**Thorax chaetotaxy**: Ac at least 25; Dc at least 30 on each side; Scts at least 20, arranged in two irregular rows; Pa at least 7, arranged in single row.

**Wing** (Fig 2A): Width: 770 μm, length/width ratio 3.96. Anal lobe rounded at base. Subcosta fading above RM area; R_1_ and R_2+3_ running close together; R_4+5_ nearly straight, M_1+2_/R_4+5_ length ratio 1.04; RM oblique; FCu placed distally of RM, VR_Cu_ 1.15. Macrotrichia observed only on wing margin.

**Legs** (Fig 2B–2G): Foreleg tibia with black, distinctly curved spur ~60 μm long, and ~30 μm long comb consisting of several distinct teeth (Fig 2B and 2C). Mid- and hindleg tibiae each bearing two spurs ~65–80 μm long, and well-separated, broad, fan-shaped combs consisting of numerous teeth ~50 μm long (Fig 2D–2G). For leg segment lengths and leg ratios, see Table 1.

**Table 1:** Leg segment lengths (in micrometres) and leg ratios of male *Eomicromimus polliciformis* sp. nov.

	fe	ti	ta_1_	ta_2_	ta_3_	ta_4_	ta_5_	LR
**p**_**1**_	1305	1420	1450	805	645	455	260	1.02
**p**_**2**_	1415	1345	850	505	400	295	215	0.63
**p**_**3**_	1415	1620	1080	690	520	345	250	0.67

fe, femur; LR, leg ratio; p_1_–p_3_, pair of legs 1–3; ta_1_–ta_5_, tarsomeres 1–5; ti, tibia

**Hypopygium** (Fig 3A–3E): Gonostylus ~185 μm long, stout, slightly arched, spatulate, narrow at base, distinctly broadened in distal part (Fig 3A). Anal point triangular, bearing dispersed scale-like spines directed posteriorly, and subapical paired structure, as shown in Fig 3B and 3D. Pseudovolsella consisting of basally merged tubercles forming trifid protrusion, each tubercle bearing seta (Fig 3C). Dorsal lobe of superior volsella directed medially, broad at base, evenly tapering to long filiform tip; ventral lobe robust, with proximal part projecting medially, distinctly bent at mid-length and directed anteriorly, apex blunt, thumb-shaped (Fig 3D and 3E). Inferior volsella stocky, with broad apex, armed with strong setae (Fig 3D and 3E).

***Eomicromimus serpens* Giłka, Zakrzewska *et* Andersen, sp. nov.** urn:lsid:zoobank.org:act:ECA1E4AB-353C-4C14-992A-545EFA029F49

(Figs 4–6)

**Derivation of the name:** In reference to a peculiar, sinuous ventral lobe of the hypopygial superior volsella, resembling a snake (in Latin, *serpens*). Noun in apposition.

**Type material:** Holotype, *CCHH 1754–13*: adult male (tarsus of left hindleg in a separate part of the same amber piece) preserved in a 14 × 10 × 6 mm cubicoid piece of Eocene Baltic amber (Fig 4A and 4B).

**Diagnosis:** Macrotrichia present on veins C, R, R_1_, R_4+5_ and on wing margin. Gonostylus straight, broadest at 1/3 length, tapering towards blunt apex. Anal point narrow and cylindrical, with sparse setae on its lateral margins. Pseudovolsella consisting of four tubercles: an anterior one close to but still separated from the cluster of the remaining three fused tubercles and placed on slightly projected ventromedian margin of gonocoxite. Ventral lobe of superior volsella narrow, sinuous, curved in different directions. Inferior volsella with apex split into paired claw-like structures.

**Description** [adult male (n = 1, holotype)]

Total body length: 5.71 mm; wing length: 3740 μm.

**Head** (Fig 4C and 4D): Eyes bare, kidney-shaped, with well-developed dorsomedian extensions. Frontal tubercles absent. Antenna with 13 distinctly separated flagellomeres (Fig 4D), AR ~2.00, plume fully developed. Length of palpomeres 3–5: ~300 μm, ~360 μm, ~425 μm. Clypeus with at least 18 setae.

**Thorax chaetotaxy** (Fig 4E): Ac at least 25; Dc at least 25 on each side; Scts over 40, mostly arranged in three/four irregular rows; Pa 7, in one row.

**Wing** (Fig 5A and 5B): Width: 855 μm, length/width ratio 4.37. Anal lobe rounded at base. Subcosta weakly visible; R_1_ and R_2+3_ parallel, running closely; R_4+5_ nearly straight, M_1+2_/R_4+5_ length ratio 1.03; RM oblique; FCu placed slightly distally of RM, VR_Cu_ 1.08. Macrotrichia observed on C, R, R_1_, R_4+5_ and wing margin.

**Legs** (Fig 5C–5H): Foreleg tibia with black, straight spur ~70 μm long, and ~40 μm long comb consisting of several distinct teeth (Fig 5C and 5D). Mid- and hindleg tibiae each bearing two spurs, ~75–80 μm long (midleg), ~90–95 μm long (hindleg), and well-separated, broad, fan-shaped combs consisted of numerous teeth ~50–60 μm long (Fig 5E–5H). For leg segment lengths and leg ratios, see Table 2.

**Table 2:** Leg segment lengths (in micrometres) and leg ratios of male *Eomicromimus serpens* sp. nov.

	fe	ti	ta_1_	ta_2_	ta_3_	ta_4_	ta_5_	LR
**p**_**1**_	1620	1670	1700	960	750	535	285	1.02
**p**_**2**_	1780	1620	990	535	410	305	205	0.61
**p**_**3**_	1795	2090	1275	780	595	395	235	0.61

fe, femur; LR, leg ratio; p_1_–p_3_, pair of legs 1–3; ta_1_–ta_5_, tarsomeres 1–5; ti, tibia

**Hypopygium** (Fig 6A–6H): Gonostylus ~230 μm long, straight, narrow at base, swollen at 1/3 length, tapering towards blunt apex (Fig 6A). Anal point narrow, cylindrical, with sparse setae on lateral margins, and subapical paired structure, as shown in Fig 6B and 6C. Pseudovolsella consisting of four tubercles: an anterior one close to, but still separated from the cluster consisting of the remaining three fused tubercles and placed on slightly projected ventromedian margin of gonocoxite, each tubercle bearing strong seta (Fig 6D); remaining setal tubercles on median margin in usual, equidistant arrangement (Fig 6A). Dorsal lobe of superior volsella directed medially, broad at base, evenly tapering to long filiform tip (Fig 6B and 6H); ventral lobe narrow, sinuous, curved in different directions relative to main body axis, as shown in Fig 6E, 6G and 6H. Inferior volsella armed with strong setae, with apex split into paired claw-like structure strongly curved dorsally, as shown in Fig 6F–6H.

***Eoriethia* Giłka, Zakrzewska *et* Andersen, gen. nov.** urn:lsid:zoobank.org:act:34567170-24D7-49DC-B423-4D1FED40B7EA

(Figs 7–10)

**Fig 7:**
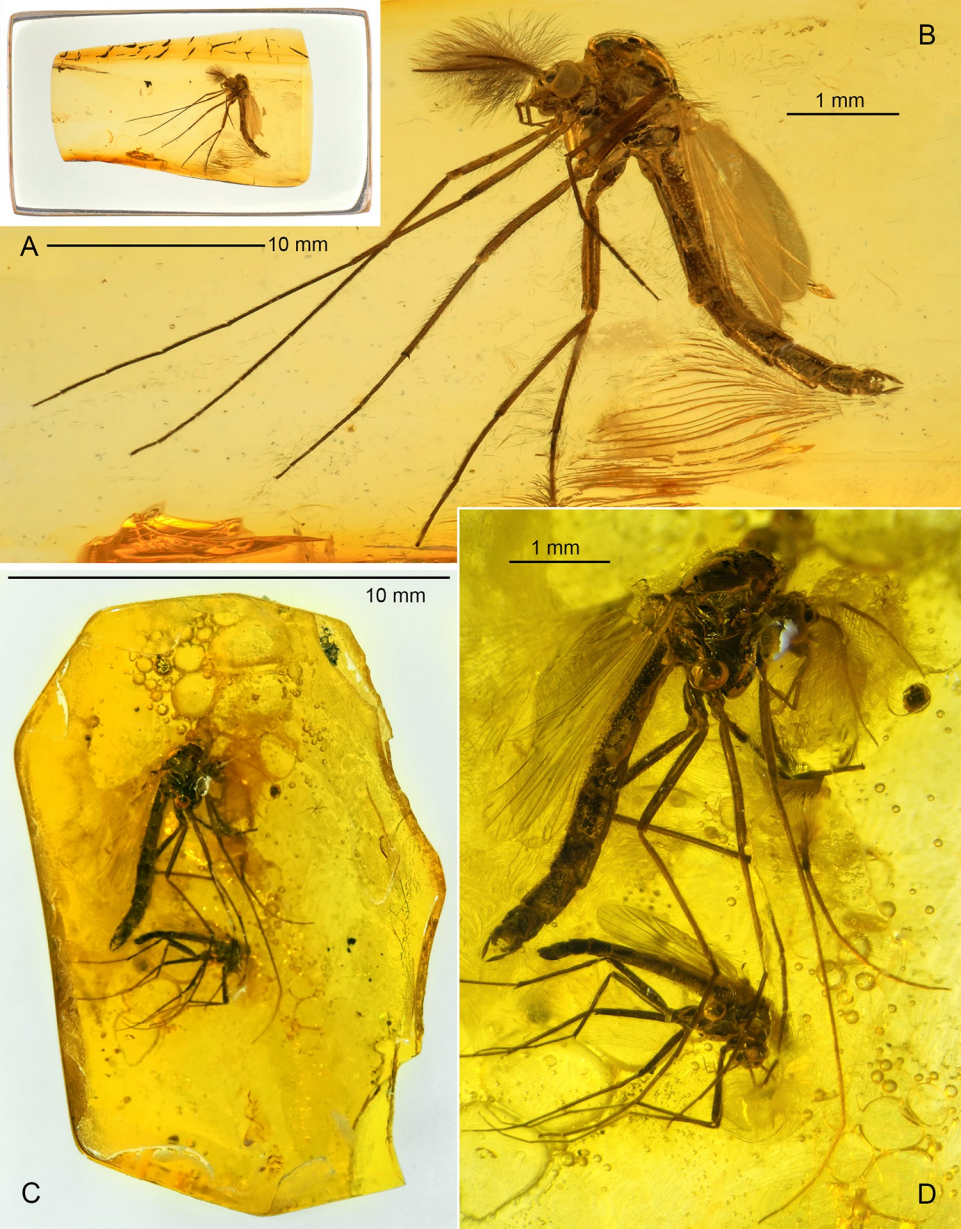
*Eoriethia ursipes* gen. *et* sp. nov., adult male. A, B, holotype (*CCHH 93–4*, Eocene Baltic amber); C, D, paratype (*CCHH 1754–5a*, larger specimen; Eocene Baltic amber). (A, C) Inclusions in amber. (B, D) Habitus.

**Fig 8:**
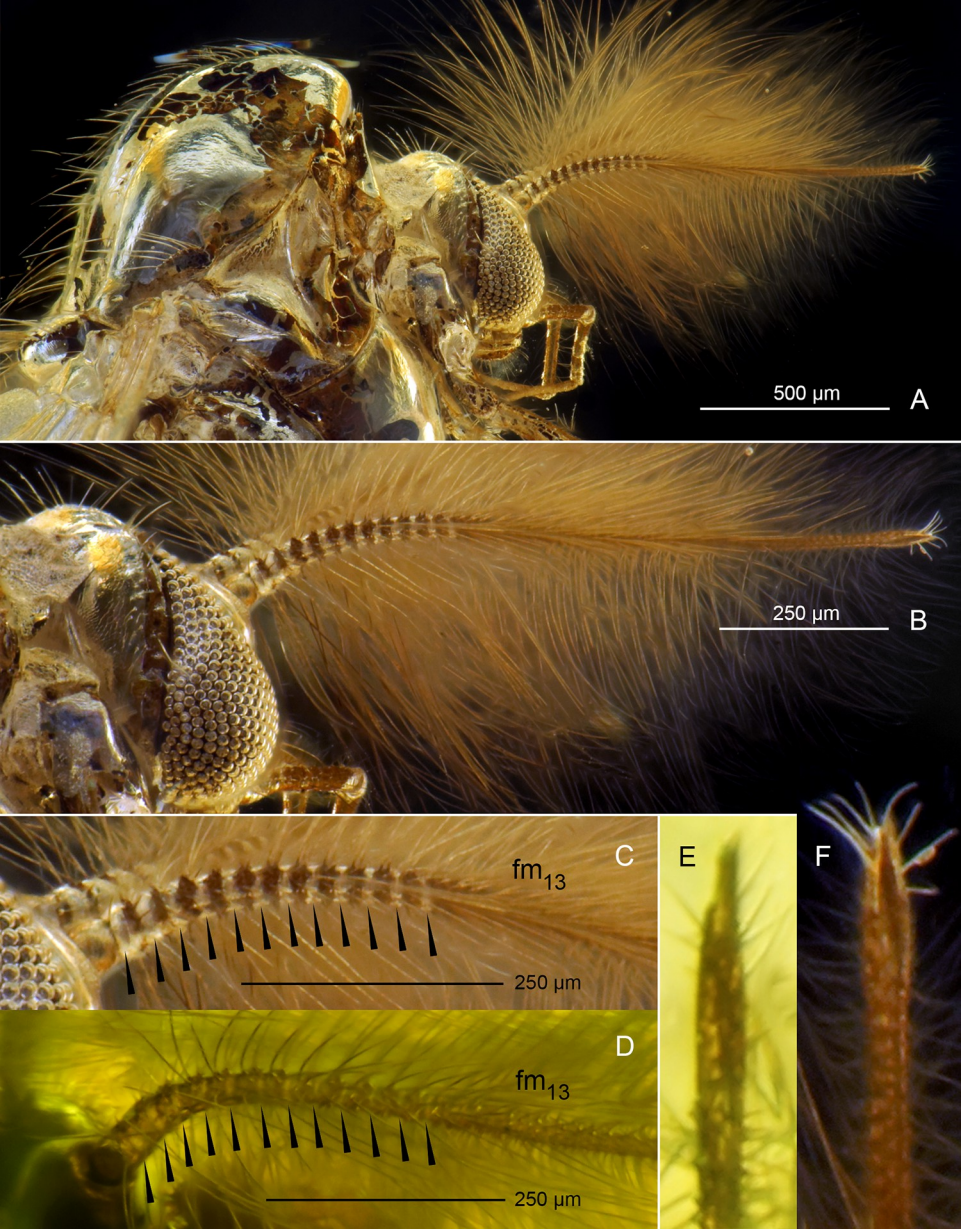
*Eoriethia ursipes* gen. *et* sp. nov., adult male. A, B, C, F, holotype (*CCHH 93–4*, Eocene Baltic amber); D, E, paratype (*CCHH 1754-5a*, Eocene Baltic amber). (A) Head and thorax. (B) Head. (C, D) Proximal part of antenna (arrowheads indicate borders between flagellomeres fm_1_–fm_13_). (E, F) Apex of ultimate flagellomere magnified.

**Fig 9:**
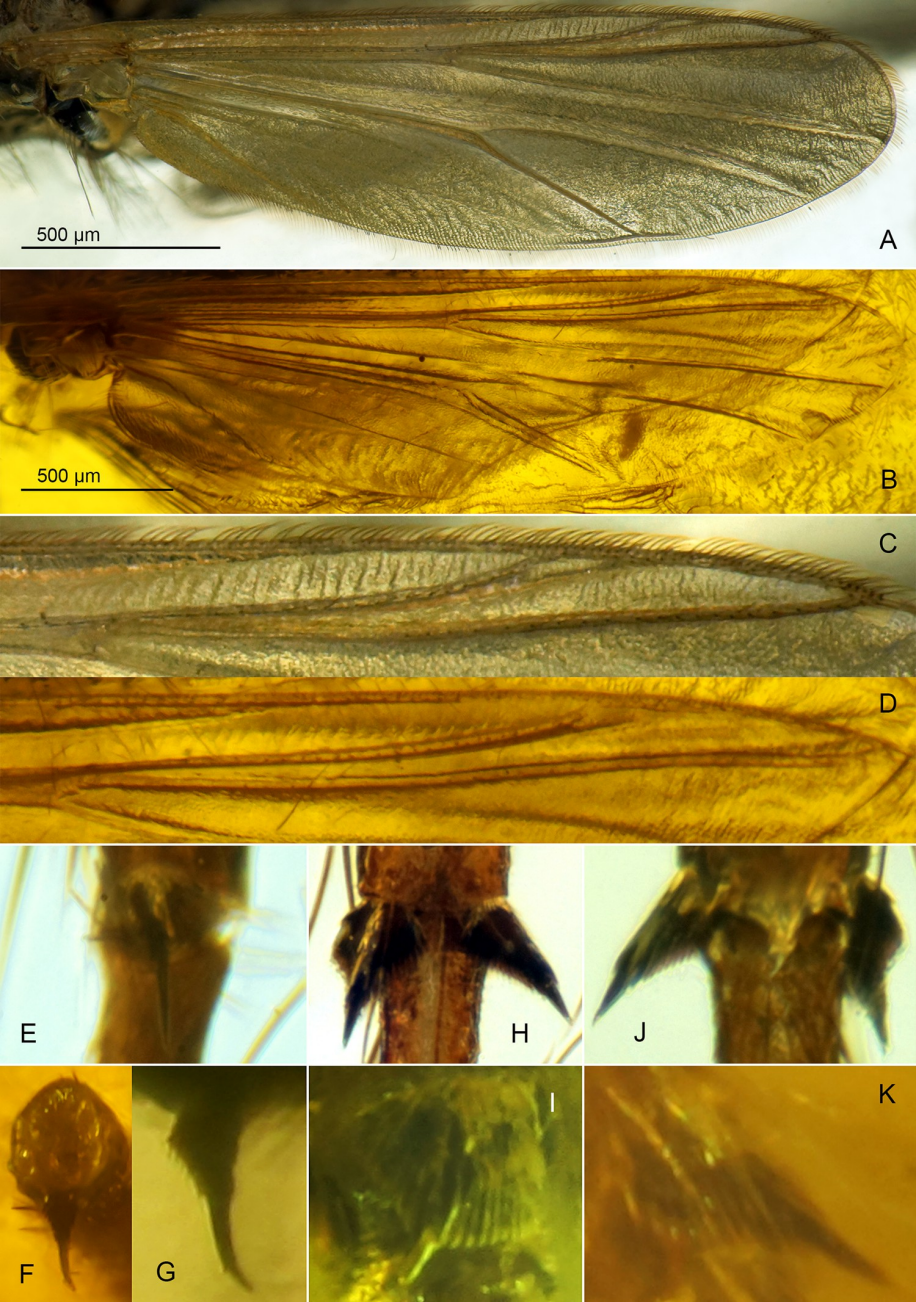
*Eoriethia ursipes* gen. *et* sp. nov., adult male. A, C, E, H, J, holotype (*CCHH 93–4*, Eocene Baltic amber); B, D, F, G, I, K, paratype (*CCHH 1754-5a*, Eocene Baltic amber). (A–D) Wing and arrangement of veins in anterior area magnified. (E–K) Tibial combs and spurs of fore (E, F, G), mid (H, I) and hind leg (J, K); G, I, K magnified ca. twice relative to E, F, H, J.

**Fig 10:**
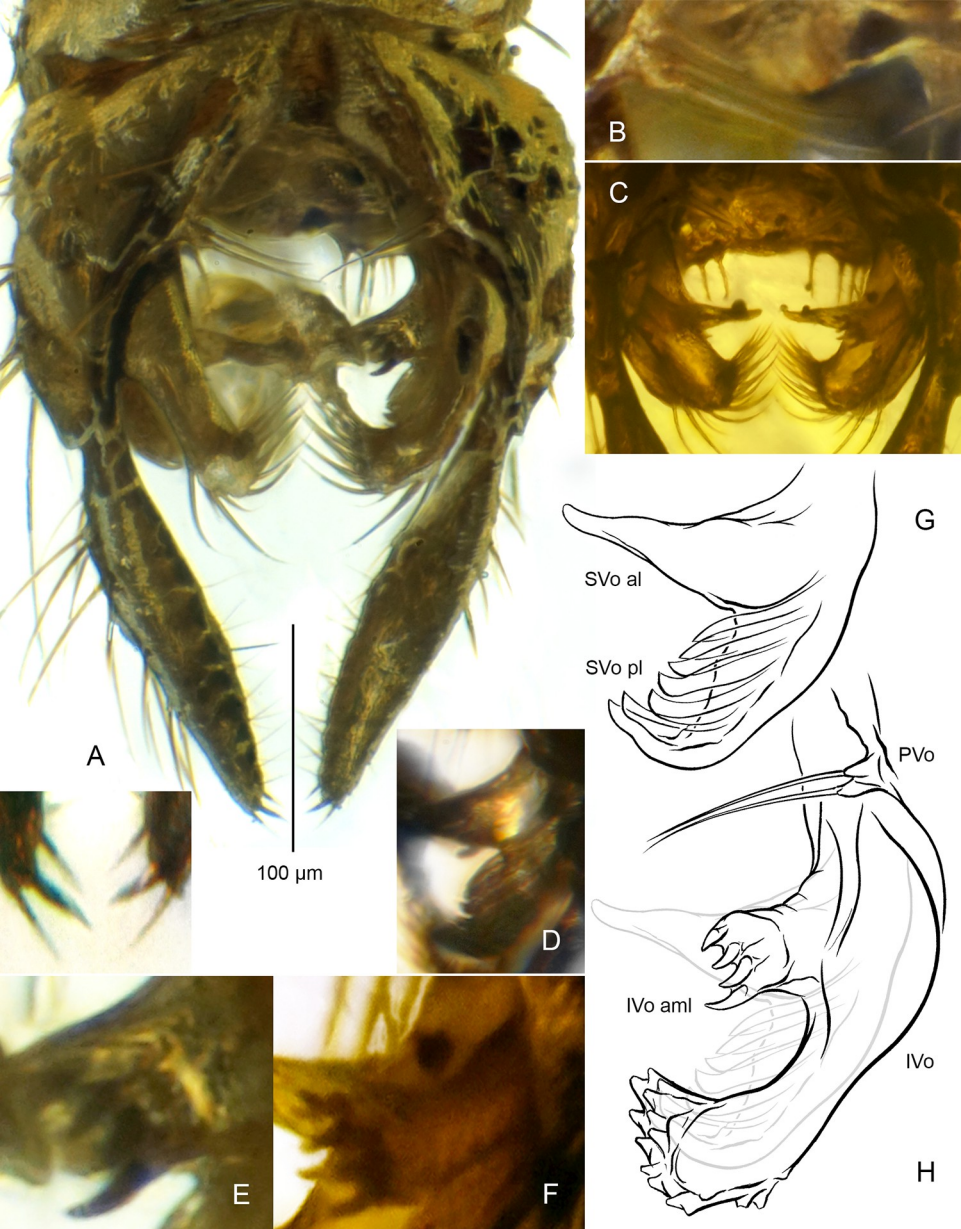
*Eoriethia ursipes* gen. *et* sp. nov., adult male. A, B, D, E, G, H, holotype (*CCHH 93–4*, Eocene Baltic amber); C, F, paratype (*CCHH 1754-5a*, Eocene Baltic amber). (A) Hypopygium in ventral aspect and apices of gonostyli magnified. (B) Pseudovolsella (PVo). (C) Arrangement of volsellae. (D, G) Superior volsella (SVo) with its anterior lobe (SVo al) and posterior lobe (SVo pl) photographed (D) and drawn (G). (E, F, H) Pseudovolsella (PVo) and inferior volsella (IVo) drawn (H), with anteromedian lobe of the latter (IVo aml) magnified on photographs (E, F).

**Type species:** *Eoriethia ursipes* Giłka, Zakrzewska *et* Andersen, **sp. nov.** (by present designation and monotypy).

**Derivation of the name:** This Eocene genus is named with reference to the compared extant *Riethia*.

**Generic diagnosis:** Eyes bare. Antenna with 13 flagellomeres. Wing squama small, with several setae. Anal point absent. Pseudovolsella in a form of merged setal tubercles. Superior volsella bilobed: anterior lobe robust, subtriangular, posterior lobe in shape of crescent keel bearing fan of strongly elongated lamelliform semi-transparent structures. Inferior volsella with prominent setal tubercles on apex; stout anteromedian lobe bearing dark, strong, claw-like spines on enlarged apex. Digitus, true median volsella and pars ventralis absent.

***Eoriethia ursipes* Giłka, Zakrzewska *et* Andersen, sp. nov.** urn:lsid:zoobank.org:act:673681CD-E503-4EE7-AACC-5ABC278A0AF1

(Figs 7–10)

**Derivation of the name:** In reference to a peculiar anteromedian lobe of the hypopygial inferior volsella, resembling a bear paw (in Latin; *ursus*—bear, *pes*—leg/paw).

**Type material:** Holotype, *CCHH 93–4*: adult male (distal part of left antenna and tarsomeres 3–5 of both hindlegs missing) preserved in a 12 × 7 × 3.5 mm piece of Eocene Baltic amber embedded in a 16.5 × 9 × 4.5 mm cubicoid piece of epoxy resin (Fig 7A and 7B). Paratype, *CCHH 1754-5a*: adult male (tarsus of left foreleg missing) preserved in a 14 × 9 × 2 mm piece of Eocene Baltic amber (Fig 7C and 7D). Syninclusions: Orthocladiinae ♂ (*CCHH 1754-5a*), Hemiptera: Aleyrodidae: Aleurodicinae ♀ in a separate part (*CCHH 1754-5b*) of the same amber piece.

**Diagnosis:** As for the genus.

**Description** [adult male (n = 2, holotype + paratype)]

Total body length: 4.50–5.26 mm; wing length: 1950–2645 μm.

**Head** (Fig 8A–8F): Eyes bare, kidney-shaped, with dorsomedian extensions. Frontal tubercles absent. Antenna with 13 flagellomeres (Fig 8C and 8D), ultimate flagellomere with distinctly narrowed tip surrounded by a crown of subapical setae (Fig 8E and 8F), AR 1.60–1.86, plume fully developed. Palp 5-segmented, basal palpomere poorly separated from head capsule, length of palpomeres 2–5: ~95 μm (n = 1), 220 μm (n = 1), 230–260 μm, 410 μm (n = 1). Clypeus with numerous, dense setae.

**Thorax chaetotaxy** (Fig 8A): Ac at least 26; Dc at least 21 on each side; Scts at least 20, stout; Pa at least 7, arranged in single row.

**Wing** (Fig 9A–9D): Broadest at mid-length, width: 600–770 μm, length/width ratio 3.25–3.44. Macrotrichia observed on C, R, R_1_ and R_4+5_. C not extended, ending well proximal to wing apex. Sc reaching C well distal of RM and FCu; R_2+3_ ending at one third between apices of R_1_ and R_4+5_; R_4+5_ nearly straight. M_1+2_/R_4+5_ length ratio 1.04–1.16; RM oblique; FCu placed slightly distal of RM, VR_Cu_ 1.07–1.11. Anal lobe rounded at base. Wing squama small, with several setae.

**Legs** (Fig 9E–9K): Foreleg tibia with black spur slightly curved at least, 45–50 μm long, and ~20 μm long comb consisting of several distinct teeth (Fig 9E–9G). Midleg tibia bearing two spurs 55–75 μm long, and combs ~35–50 μm long (Fig 9H and 9I); hindleg tibia with two spurs 65–80 μm long, and combs 50–60 μm long (Fig 9J and 9K); combs well-separated, fan-shaped, consisted of numerous teeth. For leg segment lengths and leg ratios, see Table 3.

**Table 3:** Leg segment lengths (in micrometres) and leg ratios of male *Eoriethia ursipes* sp. nov.

	fe	ti	ta_1_	ta_2_	ta_3_	ta_4_	ta_5_	LR
**p**_**1**_	990–1275	1090–1360	1145–1435	580–710	485–620	395–470	240–260	1.05–1.06
**p**_**2**_	1110–1305	1050–1305	610–740	335–455	275–360	210–250	170–275	0.57–0.58
**p**_**3**_	1100–1495	1250–1600	740–990	430–590	490 (n = 1)	315 (n = 1)	220 (n = 1)	0.59–0.62

n = 2, unless otherwise stated.

fe, femur; LR, leg ratio; p_1_–p_3_, pair of legs 1–3; ta_1_–ta_5_, tarsomeres 1–5; ti, tibia.

**Hypopygium** (Fig 10A–10H): Gonostylus 185–230 μm long, longer than gonocoxite, straight, broadest at mid-length, tapering towards blunt apex with three spine-like setae, middle seta strongest (Fig 10A). Anal point absent. Pseudovolsella placed on ventromedian margin of gonocoxite, consisting of setal tubercles merged into cluster: a distinct bifid tubercle accompanied by third slightly separate (Fig 10B). Superior volsella bilobed: anterior lobe with broad basal connection with dorsal lobe, robust, subtriangular, tapering to blunt tip directed anteromedially, posterior lobe extraordinary, consisting of crescent-shaped keel bearing fan of strongly elongated lamelliform structures with apices turned up and directed medially (Fig 10C, 10D and 10G). Inferior volsella broad at base, constricted at mid-length, distally bearing dense, prominent tubercles with strong setae (Fig 10A, 10C and 10H); anteromedian lobe of inferior volsella robust, with broad base, slightly narrowed at mid–length, bearing four dark, strong, claw-like spines on enlarged apex (Fig 10C, 10E, 10F and 10H).

## Discussion

### Terminology and morphology concepts used in diagnostics

Males of the subfamily Chironominae are known for having the most complex genital apparatus among the mime midges. Their hypopygium may be equipped with up to four pairs of highly diversified appendages bearing variously shaped setae or lamellae, and even branching onto yet further projections [15, 17]. Distinguishing the species is even more challenging when one takes intraspecific variability into account. Therefore, a proper understanding of a three-dimensional hypopygium structure, and defining homologies between its complex and minute appendages is crucial for diagnostics and phylogeny.

The complex structure of the male hypopygium, with the volsellae developed or reduced in diverse ways in the course of evolution, is still an unsolved problem in many groups of the subfamily Chironominae (cf. [16, 18]). The analysed structures, observed in fossil representatives, may hence be a source of valuable data that allow to define the probable ancestral character states (plesiomorphies). Here we present concepts concerning two hypopygium appendages: the digitus and the pseudovolsella.

### Has the true hypopygial digitus evolved in Pseudochironomini?

**Fig 11:**
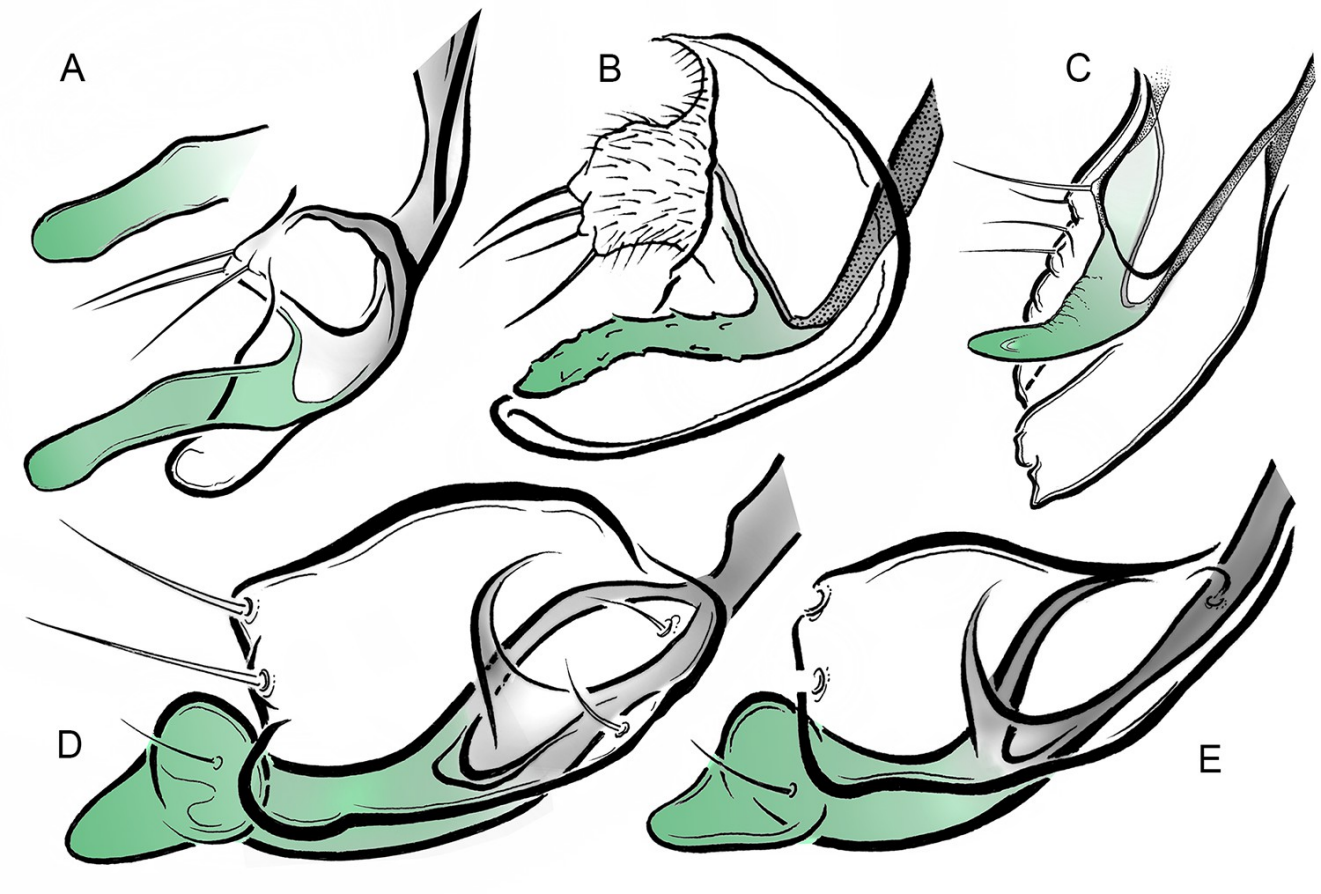
Arrangement of true digitus (green), superior volsella and lateral sternapodeme (grey) within Tanytarsini males. *Cladotanytarsus* Kieffer, 1922 (A, B), *Paratanytarsus* Thienemann *et* Bause, 1913 (C), *Tanytarsus* van der Wulp, 1874 (D, E).

By Sæther’s definition [15], the superior volsella is an apparent mesodorsal appendage, lobe or area of the male gonocoxite, while the digitus is a term that pertains to a movable finger and should only be used in reference to the ventral appendix of the superior volsella. According to this concept, the digitus cannot occur without the superior volsella; on the other hand, the digitus can be completely reduced or never-evolved, while the superior volsella is well-developed, even divided into lobes, or single-lobed and confusingly digitiform. The “true digitus” is thus typical of the tribe Tanytarsini (the majority of members of the subtribe Tanytarsina). The question is, whether, in the sense of the above definition, a homologous structure evolved in other Chironominae, including Pseudochironomini? Probably not, and below we present a set of arguments that led us to withdraw the term digitus from the concept of the hypopygium structure in Pseudochironomini males:

1. In Chironominae, the dorsoventral arrangement of the hypopygial appendages along vertical axis is as follows: superior volsella—digitus (if present)—median volsella (if present)—inferior volsella—pars ventralis (if present)—pseudovolsella (if present). The true digitus should always be situated ventral to the superior volsella, while a lobe present in some Pseudochironomini (hitherto incorrectly treated as digitus) and the superior volsella are often aligned in the same plane [16].
2. The true digitus and/or superior volsella (when the digitus is not developed) are based on a common skeleton—the lateral sternapodeme. The apodeme is usually split and/or specifically shaped (twisted) in its distal part, forming a joint-like arrangement (Fig 11), so it may indeed serve as the movable joint for the digitus, thereby supporting Sæther’s concept. The internal skeleton parts are rarely observable in specimens preserved as amber inclusions (the cuticle fixed in resin is opaque), nonetheless, we have not observed such an arrangement as described above in neither fossil nor extant Pseudochironomini males. By comparing the structures so far treated as “digitus” in extant *Riethia* Kieffer, 1917, the recently described fossil *Mesoacentron* Giłka *et al*., 2021, as well as in *Eoriethia* described here, we recognise them as median or dorso-median lobes, certainly non-movable since they are broadly fused to the inferior volsella (cf. [7, 16] and Fig 10).
3. In *Riethia*, a lobe called the digitus bears simple and/or pectinate (“moth-like”) setae or scales [16], while among Tanytarsini (here considered the only Chironominae having a true digitus) no such structures have been observed, aside from small humps or swellings on the smooth/bare surface (cf. [19, 20]), or, exceptionally, a single fine seta or minute serrations at most [21, 22] (Fig 11).

As a result, we consider the digitus an appendage that apparently has not evolved in Pseudochironomini. Admittedly, an appendage referred to as the digitus sensu Sæther was recognised as such in two of four *Manoa* Fittkau, 1963 species known to date: *M*. *tangae* Andersen *et* Sæther, 1997 and *M*. *xianjuensis* Qi *et* Lin, 2017 [23, 24]. However, these appendages could just as well be lobes of the superior volsella, having no apparent movable connection, and being placed laterally relative to the superior volsella and directed posteriorly (instead of medially or posteromedially)—an arrangement unknown in any species with true digitus. The above reasoning may support a concept of the absence of the true digitus in *Manoa*, and if so—in all Pseudochironomini.

Therefore, we abandon the use of this term in the definition of the taxa described in this work, as well in the Cretaceous *Mesoacentron* (see also the key below). Instead, we postulate the use of the term “lobe of inferior/superior volsella” within Pseudochironomini. Such unification of the terminology will prevent future confusion and improve the understanding of the character states in phylogeny.

#### A support for the term “pseudovolsella”

A discussion on the definition of the pseudovolsella, “true” median volsella, and other hypopygial appendages is ongoing. According to Sæther’s concept [15], the median volsella is a median appendage of the gonocoxite, that tends to have simple, cochleate or ramose (and many other diversely shaped) lamellae. Pinho *et al*. [18] stressed that a “typical” median volsella is originating at intermediate level between superior and inferior volsellae. Cranston [16] thoroughly explained that the structure observed among extant *Riethia* cannot be a homologue of median volsella, but an independent, stem-less structure being an aggregation formed from setal tubercles at varying stages of fusion, located on the ventral angle of the inner gonocoxite, clearly beneath the inferior volsella. For this structure he proposed the term “pseudovolsella”. Structures meeting this definition, however, hitherto referred to as “median volsella”, are also present in at least half of *Manoa* and *Pseudochironomus* Malloch, 1915 [24–26].

After re-examination of the structure in Mesozoic *Mesoacentron* (see [7]: Fig 2G and 2H and Discussion), now again observed in the Eocene tribal representatives (Figs 3C, 6D and 10B), we uphold the decision to name and treat this structure as the pseudovolsella. A degree of fusion of tubercles in Pseudochironomini is diverse and ranges from clearly separated and equidistant to completely merged into a hump or protuberance bearing setae. The number of setae, which may correlate with the number of fused tubercles, varies from one to three in extant *Manoa*, *Riethia*, Eocene *Eoriethia*, and Cretaceous *Mesoacentron*, three to four in Eocene *Eomicromimus* (with a transitional phase of the character in *E*. *serpens*—see also systematic concepts below), and a wide range of one to five setae on a prominent process in extant *Pseudochironomus*. Such a distribution of character states in different tribes of the subfamily Chironominae may indicate the structure homology, a character polarity trend, and a variable rate of its development within Chironominae lineages.

#### Systematic concepts for new taxa described

The absence of the MCu wing crossvein, along with the firmly attached to the gonocoxite, posteriorly directed gonostylus are characters that support the placement of both the new genera in the subfamily Chironominae. The oblique RM wing crossvein, the foreleg ratio close to 1.0, and, most importantly, the dark comb on the apex of the foreleg tibia justifies their position in the tribe Pseudochironomini.

The superior volsella is bilobed in both the genera described here, as well as in most of *Riethia* [16]. It is worth noting, that in recent descriptions of the three Neotropic species: *Riethia cauame* Trivinho-Strixino *et* Shimabukuro, 2018, *R*. *manauara* Neubern, Trivinho-Strixino *et* Silva, 2011 and *R*. *pantera* Trivinho-Strixino *et* Shimabukuro, 2018, a likely inaccurate understanding of an arrangement of hypopygial appendages resulted in misinterpretation of a structure, that apparently lays dorsal to the superior volsella, as a median volsella [27, 28]. Being in line with both the aforementioned dorsoventral arrangement of the hypopygial appendages, and Cranston’s concept [16], we consider this structure the dorsal lobe of a bifid superior volsella, while a true median volsella is absent.

Both the Eocene genera described here lack pars ventralis—a single or paired appendage characteristic mainly for the genus *Pseudochironomus*, the structure is also present in the Mesozoic *Mesoacentron kaluginae* Giłka, Zakrzewska, Lukashevich *et* Cranston, 2021 [7], and in a vestigial form in two *Manoa* species [23, 24].

The two new genera presented here are based on their peculiar characters, here defined as distinct generic autapomorphies, as discussed below.

#### Eomicromimus

The bilobed superior volsella, with its dorsal lobe broad at base and gradually tapering to an elongated apex and a filiform tip, along with a well-developed anal point bearing a peculiar, paired structure (generic autapomorphies), and the presence of the pseudovolsella make a unique characters’ set that supports the erection of the new genus. *Eomicromimus* includes two species described here, clearly separable by the shape of the anal point, gonostylus, ventral lobe of the superior volsella, and the inferior volsella (Figs 3 and 6). The anal point is a structure present only in some genera of the tribe: Mesozoic *Mesoacentron* and *Palaeocentron* Giłka, Zakrzewska, Lukashevich *et* Cranston, 2021, and extant *Aedokritus* Roback, 1958, *Madachironomus* Andersen, 2016 and *Megacentron* Freeman, 1961. Even though the *Eomicromimus* anal points are of different shapes in the two species described, a common feature is a peculiar, paired structure located subapically (Figs 3B and 6C). It is worth noting that a degree of fusion of the pseudovolsella varies between the two *Eomicromimus* species: in *E*. *polliciformis* it takes the form of three partially merged tubercles (Fig 3C), while in *E*. *serpens* the degree of fusion of the four tubercles is less/more advanced, with the anterior one being close to, but still separated from the cluster of remaining three, showing what we interpret as an intermediate/transitional character state (Fig 6A and 6D).

#### Eoriethia

The new genus shares a set of characters with the presumably close *Riethia*, the most relevant of which are the absence of the anal point and the pars ventralis, and the superior volsella with a tendency to be more or less split into two lobes [16, 27–29], although never in a way observed in *Eoriethia* (Fig 10, see also discussions above). A peculiarity of the superior volsella in *Eoriethia* is expressed by a distinct division into a robust anterior lobe, and particularly by the posterior lobe bearing a row of unique lamelliform structures forming a robust fan, that have not yet been observed in any Chironominae (Fig 10D and 10G). Yet another unique structure, absent in other Chironominae and incomparable with any structure among Chironomidae is the additional, stout anteromedian lobe of the inferior volsella, bearing four distinct, thick, and strongly sclerotized spines on apex (Fig 10E–10H). Both of these unusual characters, defined here as autapomorphies, led to our decision of erecting the new genus.

### Key to the identification of adult males of extinct and extant Pseudochironomini genera

1. Gonostylus directed backwards, usually rigidly connected with gonocoxite, with slight ability of flexion inwards at most (Figs 3A, 6A and 10A)… .… .… .… .… .… .…… Chironominae… 2
  - Gonostylus movable and usually folded inwards… .… .… .… .… .… .… .… .… .… .… .… .… .… .… .… .… .… .… .… .… .… .… .… .… .… .… .… .… .… .… .… .…… other Chironomidae subfamilies (not keyed)
2. Foreleg tibia with spur surrounded by darkly pigmented comb similar to those on mid- and hindlegs (Figs 2C, 5C and 9G), pars ventralis present or absent, if present—then fully developed ([7]: Fig 2E, 2G and 2H)… .… .… .… .… .… .… .… .… .… .… .… .… .… .……. Pseudochironomini… 3
  - Foreleg tibia with bristle(s) or spur at most but comb never present, pars ventralis absent or represented by depressed oval area at most (possibly a remnant of pars ventralis)… .… .…….… .… .… .… .… .… .… .… .… .… .… .… .… .… .… .… .… .… .… .… .… .. other Chironominae tribes (not keyed)
3. Anal point of hypopygium present (Figs 3A and 6A)… .… .… .… .… .… .… .… .… .… .… .… .… .… .. 4
  - Anal point of hypopygium absent (Fig 10A), anal tergite with crenate apical extension at most ([30]: Fig 10.54e)… .… .… .… .… .… .… .… .… .… .… .… .… .… .… .… .… .… .… .… .… .… .… .… .……. 9
4. True median volsella absent, pseudovolsella as aggregation/cluster of linearly merged tubercles at most (Figs 3C, 6D and 10B; [7]: Fig 2G and 2H)… .… .… .… .… .… .… .… .… .… .… .… .… .… .…… 5
  - True median volsella present ([31]: Fig 7; [32]: Fig 7; [33]: Fig 1e; [34]: Fig 1d)… .……. 7
5. Antenna with 13 flagellomeres (Figs 1Cand 4D)… .… .… .… .… .…… *Eomicromimus* **gen. nov.**
  - Antenna with 14 flagellomeres ([7]: Figs 1D and 5B)… .… .… .… .… .… .… .… .… .… .… .… .… .……. 6
6. Hypopygium with anteromedian lobe of inferior volsella, pseudovolsella and pars ventralis; anal point narrow, without spike-shaped prolongation (Fig 2E–2H); hindleg tibia without thorn-like bristles… .… .… .… .… .… .… .… .… .… .… .… .… .… .… .… .… .… .… .… . *Mesoacentron*
  - Hypopygium without anteromedian lobe of inferior volsella, pseudovolsella and pars ventralis; anal point stout, parallel-sided, with spike-shaped prolongation ([7]: Fig 7); hindleg tibia with strong thorn–like bristles arranged in row and subapical fan ([7]: Fig 6d–6g)… .… .… ..… .… .… .… .… .… .… .… .… .… .… .… .… .… .… .… .… .… .… .… .… .… .… .… .… .… .… .… .… .…… *Palaeocentron*
7. Anal point slender or sharp; antenna with 13 flagellomeres (extant species), exceptionally with 14 flagellomeres (single known fossil species, *M*. *eocenicus*) ([8]: Figs 48–50; [35]: Fig 18a)… .… .… .… .… .… .… .… .… .… .… .… .… .… .… .… .… .… .… .… .… .…… *Megacentron*
  - Anal point broad, parallel–sided or triangular/subtriangular; antenna with 13 flagellomeres ([31]: Fig 4; [32]: Fig 6; [33]: Fig 1d; [34]: Fig 1b, 1c)… .… .… .… .… .… .… .… .……. 8
8. Wing membrane with shaded areas along radial, medial and cubital veins, but without distinctly outlined spots, anal lobe moderately developed, not protruding; median volsella medially directed, with stem split apically ([32]: Figs 7, 8)… .… .… .… .… .…… *Madachironomus*
  - Wing membrane with distinct colour spots and/or crossbands, anal lobe well–developed, strongly protruding; median volsella posteriorly directed, with stem single–lobed ([31]: Figs 2, 7; [33]: Fig 1c, 1e; [34]: Fig 1a, 1d)… .… .… .… .… .… .… .… .… .… .… .… .… .… .. *Aedokritus*
9. Anal lobe of wing large, distinctly protruding; pars ventralis strongly developed ([36]: Figs 3, 4, 6)… .… .… .… .… .… .… .… .… .… .… .… .… .… .… .… .… .… .… .… .… .… .… .… . *Pseudochironomus*
  - Anal lobe of wing moderately developed and only slightly protruding at most (Fig 9B; [28]: Fig 8B); pars ventralis as small protrusion(s) at most or absent (Fig 10A; [23]: Fig 1H; [24]: Fig 6C, 6D)… .… .… .… .… .… .… .… .… .… .… .… .… .… .… .… .… .… .… .… .… .… .… .… .… .… .… .… .…… 10
10. Anteromedian lobe of inferior volsella present (Fig 10C, 10H)… .… .… .. *Eoriethia* **gen. nov.**
  - Anteromedian lobe of inferior volsella absent… .… .… .… .… .… .… .… .……. *Manoa* + *Riethia*

Note. In a recent key to the identification of Pseudochironomini males [28], a structure called “digitus” is used as a character that separates the two closely related genera, *Riethia* and *Manoa*. Given the presence/absence of the structure in members of the both genera [16, 26], and our current concept that the digitus has not evolved in Pseudochironomini—the only remaining characters that enable separation of these taxa are those of the immature stages. For this reason our key to adult males treats *Riethia* and *Manoa* jointly.
